# Functional Ablation of pRb Activates Cdk2 and Causes Antiestrogen Resistance in Human Breast Cancer Cells

**DOI:** 10.1371/journal.pone.0001256

**Published:** 2007-12-05

**Authors:** Hemant Varma, Andrew J. Skildum, Susan E. Conrad

**Affiliations:** 1 Department of Biochemistry and Molecular Biology, Michigan State University, East Lansing, Michigan, United States of America; 2 Department of Microbiology and Molecular Genetics, Michigan State University, East Lansing, Michigan, United States of America; University of Minnesota, United States of America

## Abstract

Estrogens are required for the proliferation of hormone dependent breast cancer cells, making estrogen receptor (ER) positive tumors amenable to endocrine therapies such as antiestrogens. However, resistance to these agents remains a significant cause of treatment failure. We previously demonstrated that inactivation of the retinoblastoma protein (pRb) family tumor suppressors causes antiestrogen resistance in MCF-7 cells, a widely studied model of estrogen responsive human breast cancers. In this study, we investigate the mechanism by which pRb inactivation leads to antiestrogen resistance. Cdk4 and cdk2 are two key cell cycle regulators that can phosphorylate and inactivate pRb, therefore we tested whether these kinases are required in cells lacking pRb function. pRb family members were inactivated in MCF-7 cells by expressing polyomavirus large tumor antigen (PyLT), and cdk activity was inhibited using the cdk inhibitors p16^INK4A^ and p21^Waf1/Cip1^. Cdk4 activity was no longer required in cells lacking functional pRb, while cdk2 activity was required for proliferation in both the presence and absence of pRb function. Using inducible PyLT cell lines, we further demonstrated that pRb inactivation leads to increased cyclin A expression, cdk2 activation and proliferation in antiestrogen arrested cells. These results demonstrate that antiestrogens do not inhibit cdk2 activity or proliferation of MCF-7 cells in the absence of pRb family function, and suggest that antiestrogen resistant breast cancer cells resulting from pRb pathway inactivation would be susceptible to therapies that target cdk2.

## Introduction

Approximately 40 percent of human breast tumors depend on estrogens for proliferation [1], and are therefore treated with drugs such as antiestrogens and aromatase inhibitors, which target the estrogen receptor (ER) [2]. While these therapies are very effective, the development of resistance remains an important problem that leads to relapse in many patients [2]. Multiple mechanisms have been proposed to cause acquired antiestrogen resistance in breast cancer cells, but all of these mechanisms must ultimately converge on the cell cycle machinery since antiestrogens block proliferation of these cells by affecting the cell cycle machinery [3].

Estrogens and antiestrogens control proliferation of breast cancer cells by regulating the expression of multiple components of the cell cycle machinery including cyclins D1 and A, cdc25a and the cyclin dependent kinase inhibitors p21^Waf1/Cip1^ (p21), and p27 ^Kip1^ (p27) [4], [5], [6]. These molecules regulate the activity of the cyclin dependent kinases (cdks), cdk4 and cdk2, which in turn phosphorylate and inactivate tumor suppressors of the retinoblastoma protein (pRb) family [4]. The pRb family of proteins inhibit the G1 to S phase transition by sequestering the E2F family of transcription factors [7].

The MCF-7 cell-line is the most widely studied model of estrogen dependent and antiestrogen sensitive human breast cancers [8]. MCF-7 cells were derived from a human tumor, they are ER positive (ER^+^), and their proliferation is stimulated by estrogens and inhibited by antiestrogens *in vivo* and *in vitro* [9]. They contain wild type pRb [10], and estrogen treatment leads to phosphorylation of pRb, cdk activation and progression into S phase [5], [11]. We previously investigated if pRb inactivation is sufficient to cause antiestrogen resistance in MCF-7 cells by expressing large tumor antigens (LT) of SV40 or Polyoma viruses, which bind to and inactivate pRb family proteins. We found that expression of either SV40 LT or Polyoma LT (PyLT) induced proliferation of antiestrogen arrested MCF-7 cells, and that this effect was dependent upon an intact pRb binding domain [12]. Furthermore, the ability of SV40 LT to overcome an antiestrogen-induced cell cycle arrest resided within the N-terminal 259 amino acids of the protein, excluding a role for p53 binding and a host of other functions of this large multifunctional protein [12]. A recent report using RNAi knockdown of pRb confirmed our results and extended them to *in vivo* transplants in a murine model [13]. A loss of pRb function occurs in a significant percentage (17 to 26 percent) of breast tumors [14], [15], [16], and together these results suggest that ER+, pRb negative (pRb^-^) tumors would respond poorly to treatment with antiestrogens.

In this report, we investigate the mechanism(s) by which pRb inactivation releases breast cancer cells from an antiestrogen-induced cell cycle arrest. Estrogen treatment leads to the activation of both cdk2 and cdk4 in breast cancer cell lines, and both of these kinases can phosphorylate pRb [5], [17]. We therefore investigated if these kinases are required for proliferation of MCF-7 cells in the absence of functional pRb family members. We demonstrate that cdk4 activity is required for estrogen-induced proliferation in cells with intact pRb function, but not when pRb family members are inactivated. In contrast, cdk2 activity is required irrespective of the pRb status of cells. These results indicate that cdk4 is mainly required for pRb inactivation, while cdk2 has additional targets that are required for MCF-7 cell proliferation. We also demonstrate that expression of PyLT leads to cdk2 activation, even in the presence of antiestrogens. Together, our results support a model in which cdk2 activation in response to estrogen treatment is mediated, at least in part, via pRb inactivation. They also suggest that cdk2, but not cdk4, might be a target for the treatment of ER^+^, pRb^−^ tumors that are resistant to antiestrogens or other endocrine treatments.

## Results

### pRb inactivation by PyLT induces proliferation of antiestrogen treated MCF-7 cells

**Figure 1:**
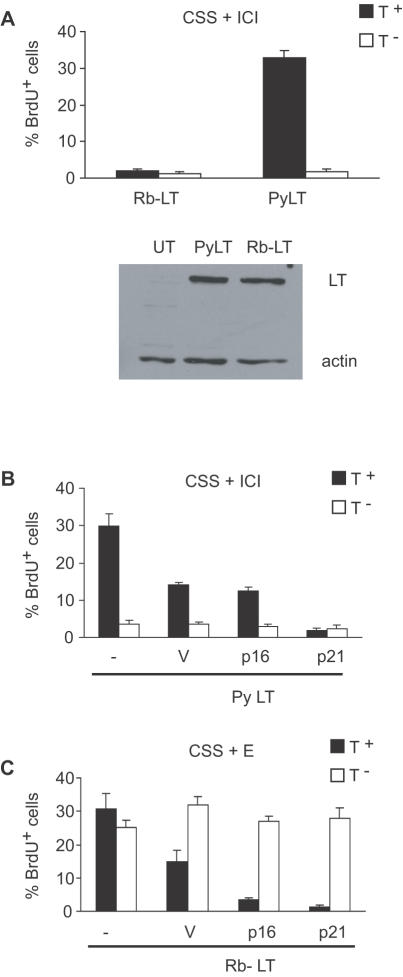
MCF-7 cells expressing PyLT are sensitive to p21 but refractory to p16 arrest. (A) MCF-7 cells were transfected with wild type PyLT or a Rb binding mutant (Rb-LT) of PyLT, treated with ICI in media supplemented with charcoal stripped serum (CSS), and proliferation was assayed by double indirect immunofluorescence for PyLT and BrdU at 48 h post transfection (Materials and Methods). The percentage of BrdU positive cells in PyLT expressing (T^+^) and non-expressing (T^-^) cells is shown. The expression of wild type and mutant PyLT in transfected cells was also assessed by immunoblotting (lower panel, UT = untransfected control). (B) MCF-7 cells were cotransfected with PyLT and either vector (V) alone, or p16/p21 encoding plasmids. Proliferation in the presence of ICI was assayed by double indirect immunofluorescence for PyLT and BrdU. Filled bars (T^+^) represent proliferation in cells expressing PyLT alone or along with vector, p16 or p21. Empty bars (T^-^) represent proliferation in cells that did not express PyLT or PyLT and cotransfected plasmid constructs. (C) The Rb binding mutant (Rb-LT) of PyLT was cotransfected with the above constructs in cycling MCF-7 cells that were treated with E, and proliferation was assayed as described above. All data shown in panels A–C is shown as mean±S.E. of at least three independent experiments, with a minimum of 100 PyLT positive and negative cells counted in each condition.

Our previous results obtained with SV40 LT indicated that the pRb binding domain of LT was required for conferring antiestrogen resistance to MCF-7 cells [12]. To confirm that this was also the case for PyLT, plasmids encoding wild type or a pRb binding mutant of LT (Rb-LT) were transiently transfected into MCF-7 cells. The cells were treated with antiestrogen ICI 182,780 (ICI) for 43 h to induce cell cycle arrest, and were then BrdU labeled for an additional 5 h before fixation to assess the percentage of cells that were actively synthesizing DNA. All experiments were conducted in phenol red–free media supplemented with charcoal stripped serum (CSS), which is serum that is depleted of estrogens [18]. The fixed cells were analyzed for PyLT expression and BrdU incorporation by indirect immunofluorescence. Since, in transient transfection experiments only a certain fraction of all cells take up transfected DNA and express detectable levels of PyLT, the percentage of cells that were actively synthesizing DNA was determined in both cells that expressed PyLT (T^+^) and those that did not (T^-^) (Figure 1A, upper panel). The results of this experiment confirmed that PyLT caused antiestrogen resistant proliferation. Furthermore, the pRb binding domain of PyLT was required for this effect, since the Rb-LT mutant was unable to increase proliferation above the levels observed in untransfected cells despite similar expression of the wild type and mutant proteins (Figure 1A, lower panel).

We also considered using another estrogen responsive human breast cancer cell line (ZR-75-1) for our experiments, but found this cell line to be much less responsive to 17β-estradiol (E) and ICI compared to the MCF-7 cells (Figure S1). These results are similar to a previous report [19]. Since MCF-7 cells are the most widely used experimental model to study the effects of E and ICI on breast cancer proliferation, and results obtained in this cell line are valid in clinical and *in vivo* models [8], we decided to use the MCF-7 cells exclusively for further investigation.

### PyLT-induced proliferation is sensitive to the cdk inhibitor p21 but resistant to p16^INK4A^

pRb is a physiological target of both cdk4 and cdk2 [17]. If the primary function of one or both of these kinases in MCF-7 cells is to phosphorylate pRb, then cells in which pRb family members are inactivated by PyLT should no longer require the activity of that kinase for proliferation. To test this possibility, MCF-7 cells were transfected with PyLT alone, or together with vector (V), p16^INK4A^ (p16) or p21 expression constructs (see Materials and Methods for details about the constructs). p16 is a specific inhibitor of cdk4, while p21 can inhibit both cdk2 and cdk4 but can also facilitate cdk4-cyclin D complex formation [7], [20]. The ability of these cdk inhibitors to block PyLT-induced proliferation of ICI treated cells was assessed by labeling transfected cells with BrdU as described above, and carrying out double indirect immunofluorescence staining for PyLT and BrdU. In a control experiment, Rb-LT was cotransfected with vector (V), p16 or p21 expression constructs into E treated cells to determine if p16 or p21 inhibited E-induced proliferation of MCF-7 in which pRb was functional. Since in transient co-transfection experiments only a fraction of the cells express the transfected genes, we assessed proliferation both in cells that expressed LT (PyLT or Rb-LT) and the cdk inhibitors (filled bars, T^+^) and those that failed to express the transfected genes (empty bars, T^-^). The results shown in Figures 1B and 1C indicate that cells transfected with the vector alone exhibited a two-fold decrease in proliferation relative to untransfected cells treated with E, or to those transfected with PyLT alone. The reasons for the decreased proliferation in the vector-transfected cells are not clear, however, the decreased proliferation was not down to the levels in ICI-treated cells. The amount of proliferation in vector co-transfected cells therefore served as the baseline to assess the effects of p16 and p21 on PyLT-induced proliferation. As shown in Figure 1B, p21 inhibited PyLT induced proliferation in ICI-treated cells, while p16 did not. However, in control co-transfections with Rb-LT, p16 inhibited E-induced proliferation as efficiently as p21 as seen by decreased BrdU incorporation in transfected cells (T^+^) compared to cells that did not express Rb-LT and p16 (T^-^) (Figure 1C). These results demonstrate that cdk4 activity is required in cells with intact pRb, but not in cells where pRb has been inactivated. In contrast, cdk2 activity is required regardless of the pRb status of cells. They also indicate that cdk4 is required mainly for pRb inactivation, while cdk2 has targets in addition to pRb. Finally, they suggest that antiestrogens do not inhibit cdk2 activity if pRb family members have been inactivated.

### Establishment of cell lines with inducible PyLT expression

**Figure 2:**
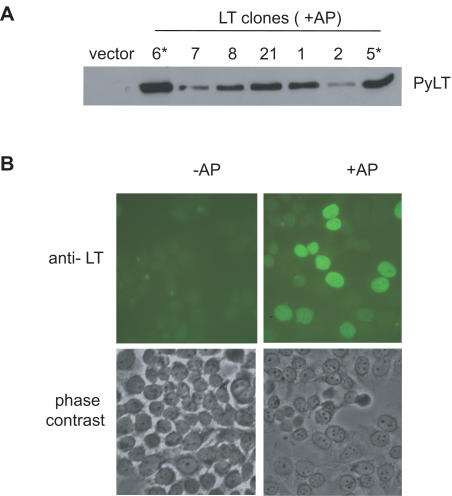
Selection of stable cell lines that express PyLT in an inducible manner. (A) Clonal cell lines stably transfected with the inducible PyLT construct were selected as described in Materials and Methods, and screened for PyLT expression by immunoblotting after 24 h of AP treatment. Asterisk denotes PyLT clones 5 and 6 that were chosen for further analysis based on robust induction. (B) One clonal cell line (LT-6) was analyzed for PyLT expression by indirect immunofluorescence. Micrographs show PyLT immunofluorescence in the absence and presence of AP for 24 h, and a phase contrast picture of the same field.

The results of the transient transfections described above indicated that cdk2 activity is required for PyLT-induced proliferation of ICI treated cells. To test if PyLT induces cdk2 activity, and to address the mechanism of its induction, we established stable inducible cell lines in which expression of PyLT was regulated by the addition of a small molecule dimerizer, AP1510 (AP). The inducible expression system and the selection of stable cell lines are described in more detail in Materials and Methods. Briefly, MCF-7 cells were transfected with a vector expressing PyLT from an AP-inducible promoter, transfectants were selected with hygromycin, and resistant clones were expanded into cell lines. The resultant clonal cell lines were screened for PyLT expression by Western blotting (Figure 2A), and several clones with low basal and robust induced levels of PyLT (LT-5 and LT-6) were chosen for further study. Expression of PyLT in the inducible cell lines was also examined by immunofluorescence, and significant variability in PyLT expression was observed in each clonal cell line upon AP treatment, ranging from background to very high levels (Figure 2B). Despite their clonal origin, only 50–60 percent of the cells expressed detectable PyLT even in continued presence of the selective agent. This variability in expression was observed for all the clonal cell lines tested (data not shown).

### PyLT induces cell cycle progression in ICI arrested cells

**Figure 3:**
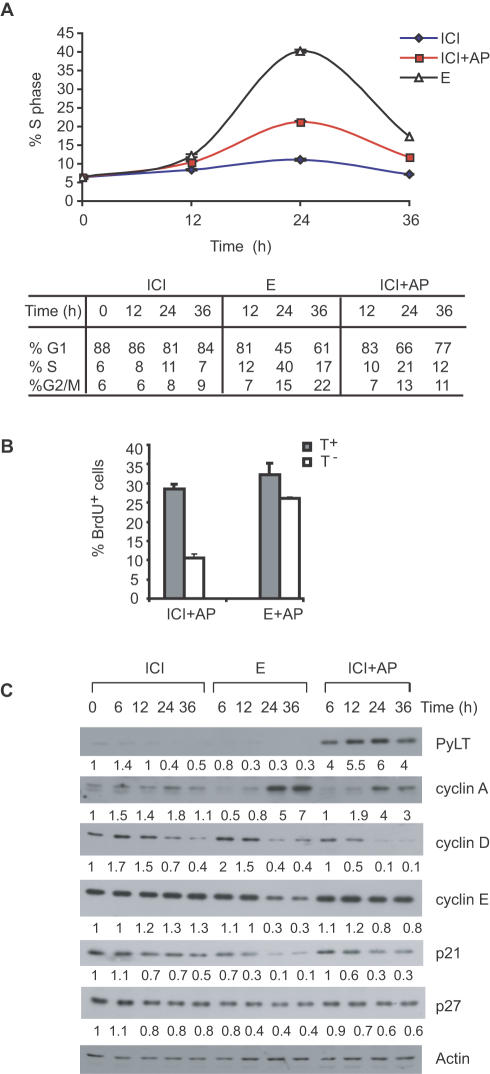
Conditional PyLT expression induces cell cycle progression, an increase in cyclin A and a decrease in p21 in ICI treated cells. (A) LT-5 cells were growth arrested in ICI for 48 h, treated with ICI, E or ICI+AP and then harvested at 12 h intervals. The cell cycle profile of each sample was analyzed by flow cytometry, and the percentage of cells in S phase is shown (top panel). The results represent the average±S.D. of one experiment done in triplicate. The percentage of cells in the different phases of the cell cycle is shown in a tabular format (lower panel). The standard deviation was typically less than 2 percent. Similar results were observed for the LT-6 clone (see Figure S2). (B) LT-6 cells were pre-arrested with ICI, treated with ICI+AP or E+AP for 24 h, and BrdU was added to cultures during the final 5 h. Cells were then fixed and stained for both PyLT and BrdU by double indirect immunofluorescence. The percentage of PyLT positive (T^+^) and negative (T^−^) cells that were also BrdU positive is shown. The results represent the average±S.D. of one experiment done in triplicate with a 100 cells counted per condition. (C) LT-5 cells were cultured, treated, and harvested in parallel to those in (A) above. Cell extracts were prepared and analyzed by western blotting for PyLT, cyclins A, E and D1 and the cdk inhibitors p21 and p27. Actin was used as a loading control. Protein levels were quantified by using the Image J software and these values were normalized to actin and are shown below the respective blots. All values are expressed relative to the zero time point that was arbitrarily assigned a value of 1.

The effects of PyLT expression on cell cycle progression after an ICI-induced G1 arrest were assessed in two independent PyLT inducible clones, LT-5 and LT-6 cells. LT-5 cells were pre-arrested with ICI for 48 h, treated with E, ICI or ICI plus AP, harvested at 12 h intervals, and analyzed by flow cytometry to determine their cell cycle phase distribution. In ICI-treated cultures, expression of PyLT increased the percentage of ICI treated cells in S phase between 12 and 24 h, from 6–7 percent at the 0 h time point to 20 percent by 24 h (Figure 3A, upper panel). As expected, the percentage of cells in G1 decreased and those in G2/M increased after PyLT induction (Figure 3A, lower panel). In comparison, up to 40 percent of cells treated with E were in S phase at the 24 h time point. Similar results were obtained with the LT-6 clone (Figure S2). These experiments demonstrate that though PyLT expression overcomes the cell cycle arrest of ICI treated cells, it is not as effective as E treatment. This was in contrast to the results in the transient transfection experiments where PyLT was as effective as E (Figure 1A and 1C). Since immunofluorescence staining indicated that only 50–60 percent of stably transfected cells expressed detectable levels of PyLT after AP treatment (Figure 2B), we hypothesized that the difference in proliferation between cultures treated with E and ICI+AP might be due to heterogeneous PyLT expression. We therefore analyzed proliferation in high and low PyLT expressing LT-6 cells. Cells were pre-arrested with ICI, then treated with ICI or E in the presence of AP, labeled with BrdU, and fixed at 24 h. Cells were stained for BrdU and PyLT, and the percentage of PyLT positive and PyLT negative cells that were also BrdU positive was determined. BrdU incorporation in cells with high PyLT expression was similar to that in E treated cells (Figure 3B), suggesting that there is a threshold of PyLT expression required to overcome an ICI-induced cell cycle arrest, and that the difference seen between the E and ICI+AP treated samples in Figure 3A is likely the result of sub-threshold PyLT expression in about half the population of cells.

### PyLT induces cyclin A and decreases p21 expression

To examine the effects of PyLT on the expression of proteins that could contribute to antiestrogen resistance, LT-5 cells harvested in parallel to those shown in Figure 3A were analyzed by immunoblotting for PyLT and for cell cycle regulators including cyclins A, E and D1, and the cdk inhibitors p21 and p27 (Figure 3C). A clear induction of PyLT was observed within 6 hours of AP addition, and high levels were maintained for the duration of the experiment. In comparison with the ICI treated cultures, there was no increase in cyclin D1 or cyclin E expression, or a major decrease in p27 expression, in response to AP treatment. However, there was an increase in cyclin A and a decrease in p21 protein levels at the 24 and 36 h time points in the ICI+AP treated cultures. These changes were also present and of greater magnitude in the E-treated cultures, and correlated with the increased percentage of cells in S phase at these time points. Similar changes in cyclin A and p21 protein were confirmed in the LT-6 clone (Figure S2). Both increased cyclin A and decreased p21 expression could contribute to cell cycle progression by increasing cdk2 activity [7]. Decreases in cyclin E and cyclin D1 protein that were observed at later time points in E-treated cells are likely a result of cell cycle progression since these cyclins are known to be degraded in late S phase [21], [22] and this correlates with an increase in the percentage of cells in S and G2/M (Figure 3A, lower panel). A decrease in cyclin D1 was observed in ICI treated cells and is likely due to the reported down regulation of cyclin D1 by ICI in MCF-7 cells [5], [11].

### PyLT induces cdk2 activity in ICI treated MCF-7 cells

**Figure 4:**
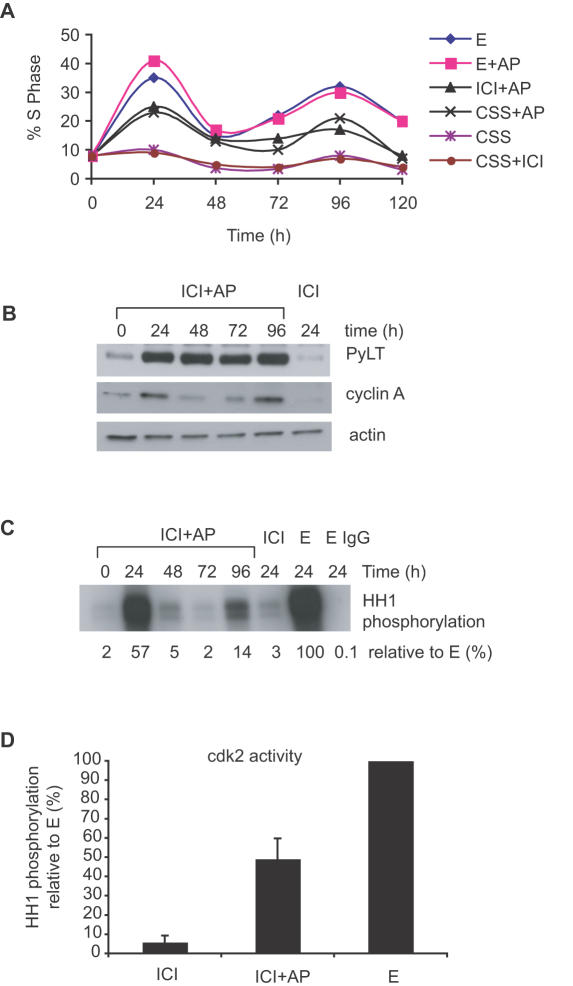
PyLT induces proliferation of ICI treated cells over 2 cell cycles and activates cdk2. (A) LT-6 cells were growth arrested in ICI for 48 h, then treated with CSS, CSS+ICI, CSS+AP, CSS+E, CSS+AP+E or CSS+ICI+AP. Cells were then harvested at 24 h intervals and the cell cycle profile was analyzed by flow cytometry. The percentages of cells in S phase at various time points are shown. The results are from one experiment, with at least 10,000 cells analyzed per sample. (B) LT-6 cells were arrested with ICI and treated with ICI+AP as described in (A). Cell extracts were prepared at the indicated time points, and analyzed for PyLT and cyclin A expression by immunoblotting. A time-matched control of cells treated with ICI alone (ICI 24 h) was included. Actin was used as a loading control. (C) LT-6 cells were growth arrested with ICI as described in (A), then PyLT was induced by ICI+AP treatment. Time matched controls (24 h) of cells incubated in ICI or E were included as negative and positive controls, respectively. Cell extracts were prepared at 24 h intervals and analyzed for cdk2 *in vitro* kinase activity using histone H1 (HH1) as a substrate. Control IgG (E 24, IgG) was used as a control for antibody specificity. The level of cdk2 activity was quantified by phosphorimager scanning and is shown below the autoradiograph. The values are relative to the E 24 h time point that was set as 100%. (D) The cdk2 activity was determined after 24 h treatment of LT-6 cells with ICI, ICI+AP or E and is expressed relative to the activity in E treated samples that was set as a 100%. The results are the average±S.D. of two independent experiments.

To further examine the mechanism(s) by which PyLT induces proliferation in ICI treated cells we carried out an extended time course experiment. LT-6 cells were pre-arrested with ICI and then treated with media containing estrogen-depleted serum (CSS), ICI or E individually, or in combination with AP. Cells were harvested at 24 h intervals for 4 to 5 days, and analyzed for cell cycle distribution and protein expression. PyLT expression caused an increase in the percentage of cells in S phase to a similar extent in both the absence of E (CSS) and presence of ICI (Figure 4A). The percentage of LT-6 cells in S phase was high in E treated cells and was not substantially increased upon PyLT induction by AP treatment. This indicates that PyLT is not able to further increase proliferation in E treated cells, and that neither AP treatment nor PyLT expression interferes with E stimulated proliferation. In all treatments, two rounds of DNA synthesis can be clearly seen, indicating that PyLT is able to promote antiestrogen resistant proliferation for at least two cell cycles. We noted that progression into the second cell cycle took longer than the first cell cycle. There are two potential contributors to this delay. First, antiestrogen treatment of MCF-7 cells causes a cell cycle arrest in the early to mid G1 [23]. Thus, cells released from an antiestrogen block (by E or PyLT) would traverse from this point in G1 phase to S phase. However to enter the S phase of second cell cycle, cells would take additional time to traverse the G2/M phase of the first cell cycle, and the complete G1 of the second cell cycle. A second contributor could be that antiestrogen arrested cells are synchronized in G1 and they progress synchronously from G1 to S phase upon release (by E or PyLT), but by the second cell cycle they enter S phase in a less synchronized manner. This could account for the prolonged but substantially lower S phase peak of the second cell cycle and is consistent with the cells entering the S phase over a prolonged period of time.

Next we directly tested the effects of PyLT expression on cdk2 activity. In a parallel experiment to that described above, ICI arrested cells were induced with AP, harvested at 24 h intervals for 4 days and subjected to western blotting and *in vitro* kinase assays for cdk2 activity. ICI and E treated cells were harvested at 24 h, and served as negative and positive controls, respectively. As shown in Figure 4B, cyclin A levels peaked at 24 and 96 h, the time points with the highest percentage of cells in S phase. Cdk2 activity was highly induced at 24 h in the ICI+AP treated cells compared to either the 0 h or the 24 h ICI treated time points (Figure 4C). However, E treated cells had approximately two-fold higher cdk2 activity (Figures 4C and 4D) at the 24 h time point than the ICI+AP treated cells, which correlated with the increased percentage of cells in S phase in these cultures (Figures 4A). Thus, PyLT expression caused an increase in cdk2 activity that closely paralleled cyclin A protein levels and S phase entry in ICI+AP treated MCF-7 cells.

## Discussion

We have demonstrated that expression of viral tumor antigens (SV40 LT and PyLT) causes antiestrogen resistance in MCF-7 cells, and that the pRb binding and inactivation domain of these proteins is required for this effect (Figure 1A) [12]. The viral LT antigens are multifunctional proteins [24], and our results cannot exclude the possibility that other functional domains contribute to antiestrogen resistant proliferation. However, LT mutants that are defective in p53 binding and truncation mutants (N terminal 1–259 and 1–136 amino acids) that lack most functional domains except the pRb inactivation domain, caused similar levels of antiestrogen resistance as full length SV40 LT or PyLT ([12] and data not shown). In addition, pRb knockdown using RNA interference causes antiestrogen resistance in breast cancer cells *in vitro* and in *vivo* [13], [25]. Together these results indicate that pRb inactivation is sufficient to cause antiestrogen resistance.

We have used the PyLT model to investigate the mechanisms by which pRb inactivation alleviates an antiestrogen-induced cell cycle arrest in MCF-7 cells. Both cdk4 and cdk2 activities are regulated by estrogen and antiestrogens in these cells [5], [11], and both can phosphorylate pRb [17] . We therefore asked whether the activity of both these kinases is required in the presence and absence of functional pRb. Using the cdk inhibitors p16 (cdk4) and p21 (cdk4 and cdk2), we confirmed that both cdk4 and cdk2 are required for 17ß-estradiol's proliferative effects in MCF-7 cells with functional pRb (Figure 1C). However, in cells where PyLT abrogated the pRb function, inhibition of cdk2 blocked proliferation while inhibition of cdk4 had no effect (Figure 1B). These results are consistent with a model in which the only requirement of cdk4 in MCF-7 cell proliferation is to inactivate pRb family members [26], [27]. Although additional tissue specific roles for cdk4 have been suggested [28], they do not appear to be required in this breast cancer cell culture model. The results that cdk2 activity is induced upon PyLT expression in ICI treated cells and cdk2 activity is required even in the absence of functional pRb (Figure 4C), support a model in which cdk2 is downstream of pRb and has additional targets [27], [29]. However, it is important to note that cdk2 is not essential for all cell cycle progression, since genetic knockout of cdk2 does not prevent somatic cell proliferation in mice [30]. Furthermore, a requirement for cdk2 activity in viral tumor antigen-induced proliferation is not universal, since both adenoviral E1A and SV40 LT were reported to overcome a cdk2 inhibitor (p27)-induced cell cycle arrest without increasing cdk2 activity [31], [32]. Although we have not tested p27's ability to inhibit proliferation in our study, others have reported that p27 inhibits proliferation of MCF-7 cells [4], [6]. The differences observed between the various systems studied could be due to differences in oncoproteins, cdk2 inhibitors, cell type, or anti-proliferative signals (serum starvation versus antiestrogen treatment) employed.

An important issue raised by our results is whether pRb inactivation is sufficient to confer complete antiestrogen resistance. In our inducible cell lines, expression of PyLT caused cell cycle progression in ICI treated cells, but the maximum percentage of cells in S phase was approximately two fold lower than in E treated cells (Figure 3A and Figure S2). Similarly, cdk2 activity was highly induced after PyLT induction, but only to approximately 50 percent of that seen in E treated cells (Figure 4D). A likely explanation for at least part of the difference between ICI+AP and E treated cells is heterogeneous PyLT expression, since only 50–60 percent cells in the inducible cell lines expressed detectable PyLT upon AP induction (Figure 2B). When only cells expressing detectable PyLT in the presence of ICI were scored for BrdU incorporation, the percentage of positive cells was similar to that seen in E treatments (Figure 3B). However, E has multiple targets in MCF-7 cells, including c-myc, p21, and cdc25A [4], [33], [34], [35]. Some of these targets may act independently of pRb [29], and disruption of their regulation may be important for maximal cdk2 activation and proliferation in the presence of antiestrogens.

A final question is the mechanism by which pRb inactivation leads to cdk activation and cell cycle progression in MCF-7 cells. Cdk2 can associate with both cyclin E and cyclin A, and the activity of both cyclin E/cdk2 and cyclin A/cdk2 complexes increased after PyLT induction in ICI treated cells (data not shown). Since the cyclin A promoter is regulated in an E2F dependent manner [36], the release of E2F upon pRb inactivation likely explains the increases in cyclin A expression (Figure 3C) and cyclin A/cdk2 activity upon PyLT induction. The mechanism of cyclin E/cdk2 activation is less clear, since cyclin E levels do not increase after PyLT expression (Figure 3C). A small but reproducible decrease in p21 expression was observed, which could increase the number of active cyclin E/cdk2 complexes. In addition, the formation of new cyclin A/cdk2 complexes could titrate both p21 and p27 away from cyclin E/cdk2 complexes, thereby leading to their activation [11].

**Figure 5:**
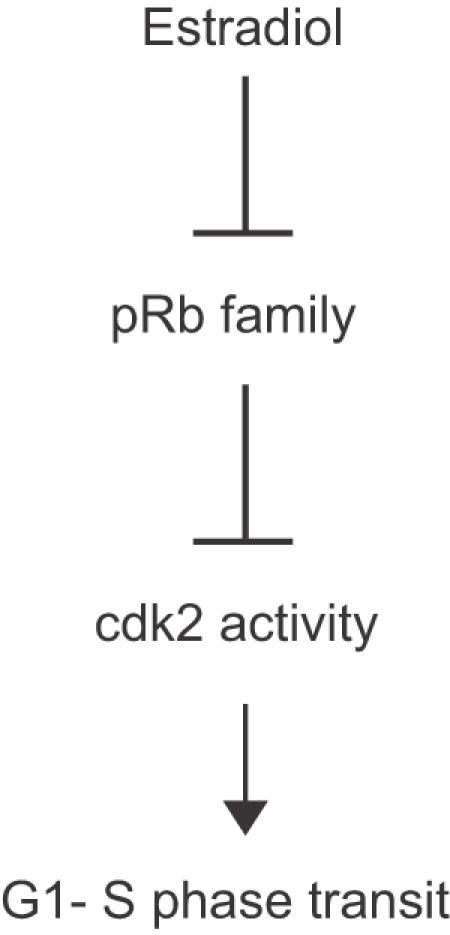
Model of E mediated proliferation of MCF-7 cells.

In summary, these results support a model in which the mitogenic effects of estrogen in breast cancer cells are largely mediated by inactivation of pRb and/or related family members, which in turn leads to cdk2 activation and cell cycle progression (Figure 5). Our result that antiestrogens do not effectively inhibit cdk2 activity or proliferation in the absence of pRb function suggests that inhibition of cdk2 activity is critical to the antiproliferative effects of antiestrogens, and this is supported by the fact that loss of either p21 or p27 causes antiestrogen resistance [6]. The Rb tumor suppressor gene is mutated in approximately 25 percent of breast cancers, and a larger percentage show alterations in the pRb pathway resulting from overexpression of cyclin D1 or loss of p16 [14], [15]. Many of these pRb^−^ tumors would likely express ER, and would therefore be treated with compounds that target ER. Our results suggest that such pRb^−^, ER^+^ tumors would be resistant to such treatments, but would retain sensitivity to pharmacological inhibitors of cdk2. These inhibitors of cdk2 that are in clinical trials [37] are potential therapies for antiestrogen resistant breast cancers with alterations in the pRb pathway.

## Materials and Methods

### Cell culture

MCF-7 cells were obtained from the Dr. M. Johnson at the Lombardi Cancer Center and ZR-75-1 cells were from the American Type Culture Collection (Manassas, VA). These cells were routinely maintained in IMEM (Biofluids) containing 5% fetal bovine serum (FBS) (HyClone), penicillin (100 units/ml) and streptomycin (100 µg/ml). Charcoal Stripped Serum (HyClone) containing media was used for some experiments. Media for PyLT inducible clones also contained hygromycin (10 µg/ml) plus G418 (50 µg/ml).

### Cell cycle analysis

Cells were fixed, stained with propidium iodide, and analyzed with a FACSVantage flow cytometer as previously described [33].

### Plasmids and cloning

Plasmids encoding the wild type polyoma large T antigen (PyLT) and pRb binding mutant (Rb-LT) were obtained from Dr. Brian Schaffhausen (Tufts University, MA) [38]. A control plasmid encoding green fluorescent protein (GFP) from a CMV promoter (Vector) and the same vector encoding p16 and p21 as GFP fusion proteins were obtained from Dr. Steve Weintraub (Washington University, MO). For inducible expression, PyLT cDNA was cloned into the pLH-Z12-I vector [33] in a two-step ligation. The PyLT cDNA was excised from the pCMV PyLT vector using *Sal*I and *Bam*HI and ligated into *Sal*I and *Bam*HI digested pIC19H vector to generate pIC19H-PyLT. In the second step, the PyLT cDNA was excised from pIC19H-PyLT using *Sal*I (site was filled in with nucleotides using T4 DNA polymerase to generate blunt ends) and *Cla*I, and ligated into the polylinker region of pLH-Z12-I that was digested with *EcoR*I (site filled in with nucleotides using T4 DNA polymerase to generate blunt ends) and *Cla*I, to generate pLH-Z12-PyLT, which was used for establishing stable cell lines.

### Construction of stably transfected cell lines expressing PyLT from an inducible promoter

A gene regulation system based on small molecule (AP1510)-regulated protein dimerization has been previously described [33] (www.ARIAD.com/regulationkits). The resultant clonal cell lines were expanded and screened for inducible PyLT expression using immunoblotting and indirect immunofluorescence.

### Chemicals and Antibodies

Chemicals used were: ICI 182,780 (A. Wakeling, Zeneca Pharmaceuticals); 17β-estradiol (E) (Sigma); 5-Bromo-2′-deoxyuridine (BrdU) (Boehringer Mannheim); and AP1510 (ARIAD Pharmaceuticals, Cambridge, MA). The rat polyclonal antibody to PyLT that was used for immunoblotting was a gift of Dr. Michele Fluck (Michigan State University, MI) and the rabbit polyclonal antibody to cdk2 was a gift of Dr. Charles J. Sherr (St. Jude Children's Research Hospital, TN). Additional antibodies used were: Anti-PyLT (Ab-1) rat monoclonal (Oncogene Science), a mouse α-BrdU antibody (Boehringer Mannheim) and FITC conjugated goat anti-rat (Sigma chemicals). Antibodies to p21, p27, actin and cyclins A, D and E, have been described previously [39].

### Immunoblotting

Immunoblotting was performed as described previously [39]. Western blots were quantified using the Image J software (National Institute of Health, Bethesda, MD).

### Indirect Immunofluorescence

MCF-7 cells were transfected with either the PyLT plasmid alone (0.3 µg) or in combination with 1 µg of pGFP (Vector), p16 or p21 as described previously [12] . After transfection, cells were treated with medium containing 5% charcoal stripped serum (CSS) and ICI (100 nM) and were labeled with 25 µM BrdU for 5 h before fixation at 48 h post transfection. The double immunofluorescence procedure for detecting transfected proteins and BrdU was described previously [12]. BrdU incorporation was detected using a mouse α-BrdU antibody [12]. PyLT was detected using the rat monoclonal anti-PyLT (Ab-1) antibody (10 µg/ml in PBS) followed by an FITC labeled goat anti-rat antibody (1∶150 dilution in 1% BSA in PBS). The percentage of PyLT expressing and non-expressing cells that were positive for BrdU was determined, with a minimum of 100 cells being counted in each condition per experiment. For the detection of PyLT expression in PyLT stable inducible cell lines, cells were treated with AP (300 nM) or vehicle and fixed 24 h after treatment. Immunofluorescence for PyLT was performed as described above. For BrdU incorporation in stable cell lines, cells were incubated in CSS+ICI (100 nM) containing media for 2 days, and then transferred to CSS+E (10 nM)+AP (300 nM) or CSS+ICI (100 nM)+AP (300 nM) containing media. After 19 h the cells were labeled with BrdU (25 µM) for 5 h, then fixed and analyzed by double indirect immunofluorescence as described above.

### Immunoprecipitations and kinase assays

Immunoprecipitations (IP) and cdk2 activity assays were performed using a modification of a published method [40] as described previously [35]. For IP experiments, 75 µg of total cellular protein lysate was incubated with specific (anti-cdk2) or control (normal goat IgG) antibodies. Kinase activity in the resulting precipitates was measured using 2 µg Histone H1 (HH1) substrate. Phosphorylated HH1 bands were quantified by phosphorimager (Molecular Dynamics).

## Supporting Information

Figure S1 Relative estrogen and antiestrogen sensitivity of two ER+ human breast cancer cell lines. MCF-7 and ZR-75-1 cells were plated in regular growth medium for 24 h and then shifted to media containing ICI (100 nM) or E (10 nM). Cells were harvested after 48 h and cell cycle analysis was performed as described in Materials and Methods. The percentage of cells in S phase is shown from one experiment performed in triplicate. Error bars represent 1 S.D.
(0.65 MB EPS)

Figure S2 Conditional PyLT expression induces cell cycle progression and an increase in cyclin A in the LT-6 clone. (A) LT-6 cells were growth arrested in ICI for 48 h, treated with CSS+ICI, CSS+E or CSS+ICI+AP and then harvested at 12 h intervals. The cell cycle profile of each sample was analyzed by flow cytometry, and the percentage of cells in S phase is shown. The results represent the average±S.D. of a single experiment done in triplicate. (B) In parallel, cells were harvested for immunoblotting and the levels of PyLT, cyclin A, p21 and actin were determined. The western blots were quantified and normalized to actin levels using Image J software. The zero h time point was arbitrarily set as 1.
(1.23 MB EPS)
